# Anti-Inflammatory Activity of Babassu Oil and Development of a Microemulsion System for Topical Delivery

**DOI:** 10.1155/2017/3647801

**Published:** 2017-12-21

**Authors:** Mysrayn Y. F. A. Reis, Simone M. dos Santos, Danielle R. Silva, Márcia V. Silva, Maria Tereza S. Correia, Daniela M. A. Ferraz Navarro, Geanne K. N. Santos, Fernando Hallwass, Otávio Bianchi, Alexandre G. Silva, Janaína V. Melo, Alessandra B. Mattos, Rafael M. Ximenes, Giovanna Machado, Karina L. A. Saraiva

**Affiliations:** ^1^Programa de Pós-Graduação em Ciências Farmacêuticas, Universidade Estadual da Paraíba, Rua Juvêncio Arruda S/N, Bairro Universitário, 58429-600 Campina Grande, PB, Brazil; ^2^Programa de Pós-Graduação em Ciências Farmacêuticas, Universidade Federal de Pernambuco, Av. Prof. Moraes Rego, No. 1235, Cidade Universitária, 50670-901 Recife, PE, Brazil; ^3^Departamento de Bioquímica, Universidade Federal de Pernambuco, Av. Prof. Moraes Rego, No. 1235, Cidade Universitária, 50670-901 Recife, PE, Brazil; ^4^Departamento de Química Fundamental, Universidade Federal de Pernambuco, Av. Prof. Moraes Rego, No. 1235, Cidade Universitária, 50670-901 Recife, PE, Brazil; ^5^Programas de Pós-Graduação em Engenharia e Ciência dos Materiais e Ciências da Saúde, Universidade de Caxias do Sul, Rua Francisco Getúlio Vargas, No. 1130, Petrópolis, 95070-560 Caxias do Sul, RS, Brazil; ^6^Departamento de Antibióticos, Universidade Federal de Pernambuco, Av. Prof. Moraes Rego, No. 1235, Cidade Universitária, 50670-901 Recife, PE, Brazil; ^7^Faculdade Boa Viagem, DeVry, Av. Jean Emile Favre, No. 422, Imbiribeira, 61200-060 Recife, PE, Brazil; ^8^Laboratório de Microscopia e Microanálise, Centro de Tecnologias Estratégicas do Nordeste, Av. Prof. Luís Freire, No. 1, Cidade Universitária, 50740-540 Recife, PE, Brazil; ^9^Núcleo de Plataformas Tecnológicas, Instituto Aggeu Magalhães, FIOCRUZ, Av. Prof. Moraes Rego S/N, Cidade Universitária, 50740-465 Recife, PE, Brazil

## Abstract

Babassu oil extraction is the main income source in nut breakers communities in northeast of Brazil. Among these communities, babassu oil is used for cooking but also medically to treat skin wounds and inflammation, and vulvovaginitis. This study aimed to evaluate the anti-inflammatory activity of babassu oil and develop a microemulsion system with babassu oil for topical delivery. Topical anti-inflammatory activity was evaluated in mice ear edema using PMA, arachidonic acid, ethyl phenylpropiolate, phenol, and capsaicin as phlogistic agents. A microemulsion system was successfully developed using a Span® 80/Kolliphor® EL ratio of 6 : 4 as the surfactant system (S), propylene glycol and water (3 : 1) as the aqueous phase (A), and babassu oil as the oil phase (O), and analyzed through conductivity, SAXS, DSC, TEM, and rheological assays. Babassu oil and lauric acid showed anti-inflammatory activity in mice ear edema, through inhibition of eicosanoid pathway and bioactive amines. The developed formulation (39% A, 12.2% O, and 48.8% S) was classified as a bicontinuous to o/w transition microemulsion that showed a Newtonian profile. The topical anti-inflammatory activity of microemulsified babassu oil was markedly increased. A new delivery system of babassu microemulsion droplet clusters was designed to enhance the therapeutic efficacy of vegetable oil.

## 1. Introduction

Babassu palm tree (*Attalea speciosa* Mart. ex Spreng, Arecaceae) is commonly found in North and Northeast regions of Brazil, mainly in the phytogeographic domain of* “Mata dos Cocais”* (Port. Lit. Palm trees forest). In its areas of occurrence, it represents the main income source for about 300.000 people in nut breakers communities in northeast region of Brazil, most of them in state of Maranhão, but also in Piauí and in Chapada do Araripe/CE. Gathering of fruits to produce oil or to sell the nuts is activities predominantly made by women, who are called* “quebradeiras de coco babaçu”* (Por. Lit. Bassabu nut breaker women) [1–3]. These communities developed autochthonous knowledge about babassu uses as source of human and animal food, utensils and tools, fuel, construction, and soil fertilizers and as cosmetic and medicines [3–6].

Among the parts used for medicinal purposes, leaves, roots, and fruits should be highlighted. Leaves and roots are used as tea for pain and wound healing, while fruits are used in a much bigger scale: mesocarp floor and milk are used for the treatment of gastritis, hepatitis, osteoporosis, skin wounds, and leukorrhea; liquid albumen is used as eyedrops to treat conjunctivitis; and the seed oil is used as laxative, vermifuge, and anti-inflammatory and for the treatment of myiasis, mycosis, skin wounds, hemorrhoids, leukorrhea and female genital inflammation, and spider bites [3–5, 7].

Vegetable oils such as olive, palm, and coconut oils are well known for their anti-inflammatory properties. Olive oil is rich in anti-inflammatory phenolics like oleocanthal and oleuropein glycosides [8, 9], while the effect of palm oil is attributed to its high tocotrienol content [10]. Like babassu oil, coconut oil is poor in phenolics and tocopherols. Its anti-inflammatory effect is assigned to the presence of lauric acid and glyceryl laurates [11].

The availability of babassu oil and its easy handling have enabled development of several types of formulations to enhance the therapeutic efficacy of the biologically active components. Targeted delivery can be achieved by applying pharmaceutical nanotechnology, which is based on the synthesis, application, and characterization of nanoscale therapeutic systems to provide a more controlled drug release. In this context, nanostructured systems, for example, microemulsions containing babassu oil, may act as new and potentially efficient therapies for benign prostatic hyperplasia due to their antiproliferative and apoptotic effects [12] and improve human immune system function by increasing superoxide anion release, phagocytosis of mononuclear phagocytes, and antimicrobial activities [13].

Microemulsion is a system of two immiscible fluids that is stabilized by an interfacial film of surfactants. It offers the advantages of spontaneous formation, thermodynamic stability, manufacturing simplicity, solubilization capacity of lipophilic and/or hydrophilic solutes, a large area per volume ratio for mass transfer, and the potential for permeation enhancement [14–17]. Thus, the aim of this work was to evaluate the topical anti-inflammatory activity of babassu oil obtained from Chapada do Araripe in Ceará (Brazil) and synthesize and characterize a microemulsion system aiming to enhance babassu oil topical anti-inflammatory activity.

## 2. Material and Methods

### 2.1. Chemicals

Phorbol 12-myristate 13-acetate (PMA), arachidonic acid (AA), ethyl phenylpropiolate (EPP), phenol, and capsaicin were purchased from Sigma Chemical Co. (St. Louis, MO, USA). The surfactants, Span 80 (sorbitan mono-oleate) and Tween® 80 (polyoxyethylene sorbitan monooleate), ethanol, 99.9% deuterated CDCl_3_, and Supelco® 37 Component FAME Mix were also obtained from Sigma Chemical Co. Kolliphor EL (polyoxyl castor oil) was from BASF SE (Ludwigshafen, Germany) and propylene glycol PA was purchased from Neon Comercial Ltda (São Paulo, Brazil).

### 2.2. Plant Material and Oil Extraction

Babassu fruits were collected from one palm tree in Araripe, located in the State of Ceará (Brazil), in July 2013. Botanical authentication was conducted by Olívia O. Cano, and a voucher specimen was deposited at the Herbarium of the Agronomic Institute of Pernambuco under number 90,472.

The oil was extracted by a well-established method that is practiced by farmers in forested areas. After harvesting, the seeds were ground with a stone grinder, and the obtained paste was mixed well and allowed to stand overnight. On the next day, cold water was added to the paste, and the upper part was collected and placed on a fire to heat it until it started boiling. Subsequently, the liquid phase was separated from the oil meal using a tissue and heated until the water completely evaporated. Finally, the oil was filtered and used [18].

### 2.3. Physicochemical Characterization and Fatty Acid Profile

Traditionally extracted babassu oil was immediately analysed for some physicochemical properties as described by Adolfo Lutz Institute [19] as follows: relative density was determined in a 10 mL glass hydrometer (pycnometer) at 25°C; refractive index at 40°C was performed using a Abbé refractometer; peroxide values were calculated from the iodine release from potassium iodide and expressed as milliequivalents of active oxygen/kg of oil; rancidity (lipid oxidation) was determined by Kreis reaction using phloroglucinol in acid medium; acid values were expressed as milligrams of KOH required to neutralize the free acids of 1 g of oil, which was determined by titration in methanolic solution.

The transesterification procedure of the oil was realized in accord with Metcalfe et al. [20]. The oil (150 mg) was mixed with 4.0 mL of NaOH in 0.50 M methanol and incubated at 100°C for 5 minutes. Next, 5.0 mL solution of BF3 (12%) in methanol was added in the mixture and was heated for 2 minutes. After cooling to room temperature, 5.0 mL of a saturated sodium chloride solution was added. The mixture was transferred to a separation funnel containing 20.0 mL of petroleum ether and vigorously stirred. After a period of rest, the aqueous phase was discarded and the ether phase was filtered. The solvent was evaporated in a rotary evaporator at 60°C and the residual solvent was removed with nitrogen flow. Methyl esters were solubilized in n-heptane before injection into the gas chromatographer [21].

The samples were analyzed using an Agilent Technologies (Palo Alto, CA, USA) 5975C single quadrupole GC-MS equipped with a nonpolar HP-5MS (Agilent) fused silica capillary column (30 m × 0.25 mm i.d.; film thickness 0.25 mm). The oven was initially held at 150°C for 2 min, increased to 230°C by 5°C/min (held for 7 min), and finally increased to 260°C by 4°C/min. The final temperature was maintained for 7.5 min. The carrier gas was helium supplied at a constant flow of 1 mL/min and the split/splitless injector was maintained at 230°C. The applied ionization potential was 70 eV; the scan range was from 35 to 450 *m*/*z* with scan rate of 0.5 scans/s. A standard fatty acid methyl ester mixture (Supelco 37 Component FAME Mix, USA) was used to identify the fatty acid methyl esters. Fatty acid data were expressed as percentage of total peak area.

### 2.4. NMR Analysis

The extracted oil (approximately 50 mg) was dissolved in 0.6 mL of CDCl_3_ and placed in a 5 mm NMR tube. The NMR analyses were performed on a Varian 400-VNMRS (Agilent Technologies, CA, USA) at 26°C, operating at the frequencies of 399.74 and 100.51 MHz for ^1^H and ^13^C, respectively. ^1^H NMR spectra were recorded with 2.5 s acquisition times, sweep widths of 6.4 kHz, 45° pulse angles, and 1 s delay times. ^13^C{^1^H} NMR spectra were recorded with 3.04 s acquisition times, sweep widths of 21.5 kHz, 45° pulse angles, and 1 s delay times. For processing ^13^C spectra, zero filling and line broadening of 1 Hz were applied prior to Fourier transformation. The chemical shift scale was indirectly referred to tetramethylsilane (TMS) by the residual CHCl_3_ signal at 7.26 ppm for ^1^H spectra and the signal at 77.0 ppm for ^13^C spectra. 

### 2.5. Topical Anti-Inflammatory Activity

#### 2.5.1. Animals

Male Swiss and BALB/c mice (25–30 g, *n* = 6) were provided by the Animal Facility of Federal University of Pernambuco. The animals were housed and kept in a room with controlled temperature (23 ± 2°C) under a 12/12 h light/dark cycle with food and water ad libitum. Experiments were conducted according to the Guide for the Care and Use of Laboratory Animals from the US Department of Health and Human Services. The project had been previously approved by the Animal's Ethics Committee of Federal University of Pernambuco (number 23076.039887/2014-51).

#### 2.5.2. Ear Edema Measurement

For evaluation of ear weight, mice were euthanized and 6 mm diameter samples were taken from both ears using a biopsy punch (Richter®, Brazil). Each biopsy was weighed on a semi-micro analytical balance (AUW-D 220, Shimadzu, Japan). Ear edema (EE) was expressed as the increase in ear sample weight, using the following formula:

(1) $$\begin{array}{c} EE\;\left(\;mg\;\right)=wRE-wLE, \end{array}$$

where *w*RE is the weight obtained from the right ear sample (inflamed ear) and *w*LE is the weight obtained from the left ear sample (noninflamed ear).

For all treatments, animals were anesthetized with 1% halothane. Right ears were then challenged with different phlogistic agents diluted in acetone (20 *μ*L). Babassu oil (1, 3, and 10 *μ*L/ear) was applied topically in 20 *μ*L acetone, while microemulsion was applied with no dilution. Dexamethasone or indomethacin (0.1 and 0.5 mg/ear, resp.) was used topically as a positive control. Ruthenium red (3 mg/kg, s.c.) was used as a positive control for capsaicin-induced ear edema.

#### 2.5.3. PMA-Induced Ear Edema

Ear edema was induced by topical application of 20 *μ*L of PMA (2.5 *μ*g/ear) in acetone on both sides of the right ear. Left ears received only acetone as a control. After 15 min, 10 *μ*L of babassu oil (100 and 12.2%), babassu microemulsion, microemulsion vehicle (48.8% surfactants, 39% aqueous phase, and 12.2% water), 0.5% dexamethasone, or acetone were applied to both sides of the right and left ears. Six hours after PMA administration, mice were euthanized for ear edema measurement [22, 23]. To avoid misinterpretation of the results due to a possible barrier effect of babassu oil on ear surfaces, an oral treatment with babassu oil was also evaluated against PMA-induced ear edema. Shortly, mice were treated with babassu oil (100, 300, and 1000 mg/kg) or indomethacin (10 mg/kg, p.o.) 60 min before PMA administration [23].

#### 2.5.4. Investigation of Babassu Oil Mode of Action on Ear Edema

For elucidation of the mechanisms underlying the topical anti-inflammatory activity of babassu oil on PMA-induced mice ear edema, different phlogistic agents were used to induce ear edema: arachidonic acid (2 mg/ear), ethyl phenylpropiolate 5% (20 *μ*L/ear), phenol 10% (20 *μ*L/ear), and capsaicin (0.25 mg/ear). Immediately after acetone evaporation, babassu oil (10 *μ*L/ear) or indomethacin (0.5 mg/ear)/dexamethasone (0.1 mg/ear) were topically applied on both sides of the right ear, while the left ear received 20 *μ*L of the vehicle. Ruthenium red (3 mg/kg, s.c.) was used as positive control for capsaicin-induced ear edema and it was administered 30 min before capsaicin.

After 1 h, mice were euthanized for ear edema measurement. Mice challenged with capsaicin were euthanized after 30 min of exposure. AA-induced ear edema was performed in BALB/c mice [23–25].

### 2.6. Preparation of Emulsions

A series of oil-in-water emulsions with HLB values ranging from 4.5 to 15 was prepared with the surfactants Span 80 and Tween 80 at a 2% total blend concentration w/v, 93% water, and 5% babassu oil using the Griffin equation [26]. The aqueous phase (water plus Tween 80) and the oil phase (oil plus Span 80) were heated to 70 ± 5°C separately. Both phases were mixed by the inversion method with mechanical stirring (9.000 rpm) for 5 min in ULTRA-TURRAX homogenizer equipment (Unique/DES500, São Paulo, Brazil). The samples were characterized 24 h after preparation.

### 2.7. Droplet Size Analysis by Dynamic Light Scattering (DLS)

The droplet size distribution of the dispersed phase of the emulsions was determined by DLS using the Nanotrac Wave equipment (Microtrac Inc., Montgomeryville, PA, USA) with measurement capability from 0.8 to 6500 nanometers. The data were calculated using the manufacturer's software. Each emulsion was diluted with aqueous propylene glycol (1 : 100) and analyzed in triplicate.

### 2.8. Turbidimetric Method

The emulsions were diluted 1 : 25 with aqueous propylene glycol, and the percentage of transmission (%  *T*) was measured at 600 nm using a spectrophotometer (UV-mini 1240, Shimadzu, Japan). With the blank control set at 100% transmission, the turbidity of the diluted emulsion was calculated as follows: turbidity = 100 − %*T* [27]. The reported results were the average of three determinations.

### 2.9. Construction of the Pseudo-Ternary Phase Diagram

Babassu oil, Span 80, and Kolliphor EL were selected as the oil phase and surfactants, respectively. Surfactant mixtures were tested at a ratio of 6 : 4 (w/w), as defined by the Griffin equation. The pseudo-ternary phase diagrams were constructed using the water titration method at room temperature, and the results were plotted using Software Origin® Pro 8.0. For each phase diagram, mixtures of surfactants and oil were prepared at weight ratios of 1 : 9, 2 : 8, 3 : 7, 4 : 6, 5 : 5, 6 : 4, 7 : 3, 8 : 2, and 9 : 1 (%_w/w_). These mixtures were titrated dropwise with water-propylene glycol (1 : 3) under mechanic stirring. After equilibration, the systems were visually characterized. Single-phase, transparent mixtures were designated as microemulsions [28].

After data analyses of the pseudo-ternary phase diagrams, the microemulsion system was prepared by mixing babassu oil (12.2%) with the surfactants (48.8%) before adding the aqueous phase (39%) under magnetic stirring. After two days, the system was evaluated by complementary techniques, as described below.

### 2.10. Characterization of the Microemulsion

#### 2.10.1. Electrical Conductivity (EC)

EC was evaluated using a digital conductivity meter (mCA 150P, Tecnopon, São Paulo, Brazil) previously calibrated with a calibration solution (146.9 *μ*S/cm). The electrode was inserted directly into 10 mL of the formulation. The analysis was performed in triplicate at a temperature of 25 ± 0.5°C.

#### 2.10.2. TEM

The sample was dropped onto a 300 mesh carbon-coated cooper grid and negatively stained with 2% phosphotungstic acid. The grid was analyzed in an FEI Tecnai Spirit Biotwin G^2^ microscope (Hillsboro, Oregon, USA) operated with 80 KV of accelerating voltage.

#### 2.10.3. SAXS

SAXS experiments were performed on the SAXS1 beamline of the Brazilian Synchrotron Light Laboratory (LNLS, São Paulo, Brazil), monitored with a photomultiplier, and detected on a Pilatus detector (300k Dectris) positioned at 836 mm that generated scattering wave vectors (*q*) from 0.13 to 2.5 nm^−1^. The wavelength of the incident X-ray beam (*λ*) was 0.155 nm^−1^. Background and parasitic scattering were determined by separate measurements on an empty holder and subtracted from the scattering data of the sample. The X-ray scattering was experimentally determined as a function of the scattering vector, *q*, whose modulus is given as follows:

(2) $$\begin{array}{c} q=\frac{\left(4\pi \;sen\;\theta \right)}{\lambda }, \end{array}$$

where *θ* is half of the scattering angle (2*θ*).

#### 2.10.4. DSC

DSC analysis was performed using a Shimadzu DSC 50 (Kyoto, Japan) with 9-10 mg of sample under a nitrogen atmosphere with a flow rate of 50 mL/min. The melting temperature and enthalpy were calibrated with indium and zinc standards. The samples were analyzed from −50 to 60°C with a heating rate of 10°C/min.

#### 2.10.5. Rheological Analysis

The rheological behavior of the formulation was investigated on an Anton Paar Physica MCR502 oscillatory rheometer (Ashland, VA, USA) with cone and plate geometry (50 mm diameter). The gap between the cone and plate was set at 0.05 mm. The measurements were taken at 25°C over a shear rate (*γ*) range from 0.1 to 1000/s.

### 2.11. Statistical Analysis

Results were expressed as mean ± SEM and normal distributions were checked by Shapiro-Wilk test. Then, data were analyzed by one-way ANOVA followed by Tukey's test using GraphPad Prism® 5.0 with significance set at *p* < 0.05.

## 3. Results and Discussion

### 3.1. Physicochemical Characterization, GC-MS, and NMR Analyses of Babassu Oil

Physicochemical parameters of babassu oil and reference values are placed in Table 1, while fatty acid composition obtained by GC-MS is showed in Table 2. The main fatty acids found were dodecanoic acid (lauric acid) (40.78%), 9-octadecenoic acid (oleic acid) (21.35%), tetradecanoic acid (myristic acid) (20.05%), and hexadecanoic acid (palmitic acid) (12.26%) (see SM Figure 2). These data are in accordance with previous published data and with reference standards of Brazilian regulatory agencies [29–31]. Differences in relative density and refractive index from reference values can be explained since reference values are derived from refined oils. Nevertheless, those parameters indicative of oil degradation, as acidity, rancidity, and peroxide value, are well below the established limits of quality.

^1^H NMR and ^13^C NMR analyses were performed to characterize the components of the sample and determine the distribution of fatty acids in the glycerol backbone of babassu oil (Table 3) (see SM Figures 3 and 4). NMR signals were assigned according to the literature [31–35]. There are some advantages to using NMR spectroscopy to study fatty acid compositions: (a) the ability to directly operate on the oil sample without any chemical manipulation, and (b) NMR is the only direct instrumental method by which the positional distribution of fatty acids on glycerol can be specifically identified [36].

The signals at 5.33, 2.01, and 2.74 ppm were assigned to the olefinic hydrogens, the protons attached to the allylic carbons, and the protons attached to the* bis*-allylic carbons, respectively (Table 3). These signals revealed the presence of unsaturated fatty acids, such as oleic acid in the oil mixture. Moreover, the last signal indicated that a small amount of linoleic acid was present. Linolenic acid was not detected in this mixture because no signal was observed at 0.98 ppm, which corresponds to the terminal methyl group of the fatty acid [32]. These results agree with those obtained in the GC-MS spectra (Table 2). The full identification of every fatty acid component was not possible due to signal overlap in the spectra.

^13^C NMR spectra provide information regarding the positional isomerism of fatty acids in the glycerol backbones of triacylglycerols (TAG), diacylglycerols (DAG), and monoacylglycerols (MAG) [35]. When analyzing the spectral region of the carbonyl group, only two signals at 173.2 and 172.8 ppm were observed (Table 3). This pattern indicated that the oil mixture includes only TAG, a fatty acid distributed at the sn-1,3 and sn-2 positions. Moreover, the spectral region of the acyl carbons between 60 and 73 ppm shows only two peaks (68.85 and 62.07 ppm) (Table 3), supporting that the sample only contains TAG isomers. The presence of unsaturated fatty acids that was observed in the ^1^H spectrum was confirmed in the ^13^C spectrum by the signals between 128.00 and 130.00 ppm. This region is characteristic of ethylenic carbons. The chemical shifts at 129.66 and 129.99 ppm indicated that the unsaturated fatty acids in the oil mixture were majority oleic acid, in agreement with the GC-MS results.

### 3.2. Topical Anti-Inflammatory Activity

PMA-induced ear edema is a useful model for screening topical anti-inflammatory compounds and/or plant extracts that act at a variety of levels. Skin inflammation induced by topical PMA administration is mediated trough protein kinase C (PKC) activation of NF-*κ*B, with production of tumor necrosis factor-*α* (TNF-*α*), cyclooxygenase-2 (COX-2), and prostaglandin E2 (PGE2) [37–39]. PMA given to mouse ears induces mast cell infiltration with release of mediators that increase vascular permeability and promote neutrophil influx [40, 41]. Anti-inflammatory efficacy of evaluated compounds implies mainly interference of arachidonic acid metabolism, since phospholipase A2 (PLA2), COX, and lipoxygenase (LOX) inhibitors have been shown to inhibit PMA-induced inflammation [23, 42].

**Figure 1:**
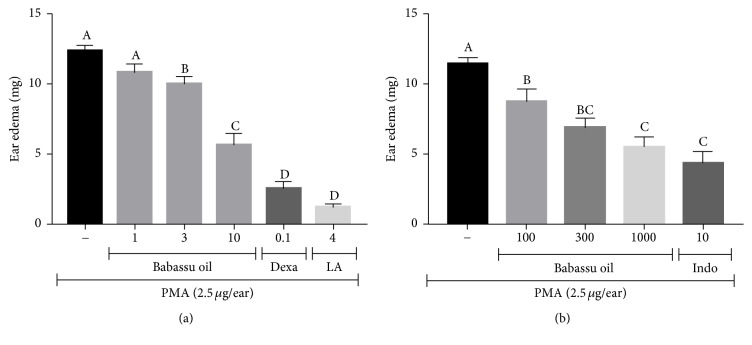
(a) Topical and (b) systemic anti-inflammatory activity of babassu oil in PMA-induced ear edema. Results are expressed as the mean ± SEM and analyzed by ANOVA followed by Tukey's test with *p* set at 0.05. Different letters show statistical differences between groups (*p* < 0.05). (−) Negative control (acetone); babassu oil (*μ*L/ear in (a)) and (mg/kg, p.o. in (b)); (LA) lauric acid (mg/ear); (Dexa) dexamethasone (mg/ear), and (Indo) indomethacin (mg/kg, p.o.).

Babassu oil (3 and 10 *μ*L/ear) was able to inhibit PMA-induced ear edema in 19.1 (*p* < 0.05) and 54.1% (*p* < 0.001), respectively. Lauric acid (4 mg/ear), the major fatty acid of babassu oil, showed 90.3% of inhibition (*p* < 0.001), while dexamethasone inhibited the ear edema in 79.7% (*p* < 0.001) (Figure 1(a)). To avoid misinterpretation of the results due to a possible barrier effect of babassu oil on ear surfaces, an oral treatment with babassu oil was also evaluated against PMA-induced ear edema. When given orally, babassu oil (100, 300, and 1000 mg/kg) showed 23.5 (*p* < 0.05), 39.7, and 51.9% of edema inhibition (*p* < 0.001), while indomethacin (10 mg/kg) inhibited the edema in 62.0% (*p* < 0.001) (Figure 1(b)).

It is worth to comment that the high content of lauric acid in babassu oil defines the importance of this study, since its antibacterial and anti-inflammatory activities are described in the literature [43–45], through inhibition of MAPK pathway and subsequent NF-*κ*B activation, which are prominent contributors to the production of proinflammatory cytokines and chronic inflammatory responses [46], including in PMA-induced ear edema model. After topical administration, lauric acid penetrates the skin and accumulates in the dermis [47].

**Figure 2:**
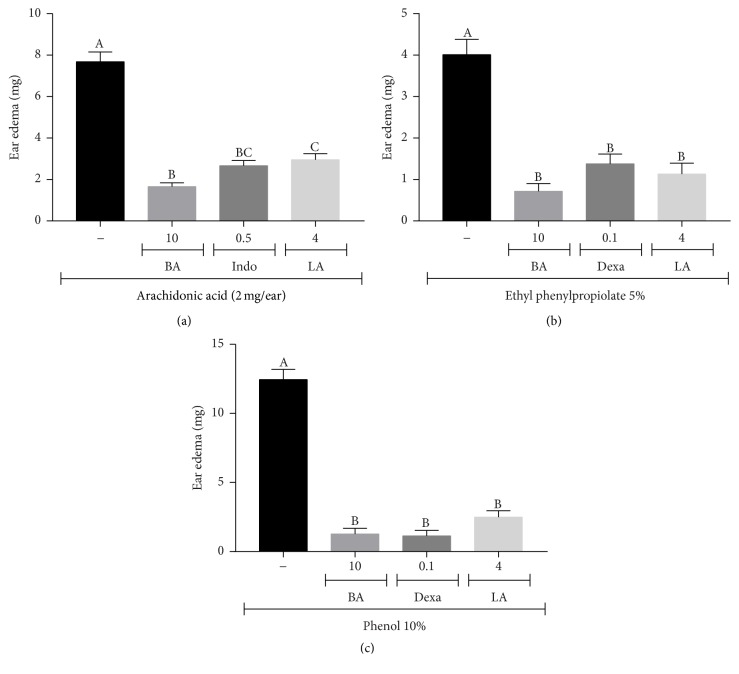
Topical anti-inflammatory activity of babassu oil and lauric acid in (a) arachidonic acid; (b) ethyl phenylpropiolate; and (c) phenol-induced ear edema. Results are expressed as the mean ± SEM and analyzed by ANOVA followed by Tukey's test with *p* set at 0.05. Different letters show statistical differences between groups (*p* < 0.05). (−) Negative control (acetone); (BA) babassu oil (*μ*L/ear); (LA) lauric acid (mg/ear); (Indo) indomethacin (mg/ear); and (Dexa) dexamethasone (mg/ear).

To further elucidate the topical anti-inflammatory activity of babassu oil, different phlogistic agents-induced ear edema was performed. Arachidonic acid-induced ear edema is a useful tool to identify compounds that interfere in eicosanoid pathway, such as COX and LOX inhibitors. However, this model is not sensitive to PLA2 inhibitors such as glucocorticoids [23, 48–50]. Here, both babassu oil (10 *μ*L/ear) and lauric acid (4 mg/ear) inhibited AA-induced ear edema by 78.5 and 61.6%, respectively (*p* < 0.001). Indomethacin (0.5 mg/ear), a nonselective inhibitor of COX-1/2, decreased the ear edema by 65.4% (*p* < 0.001) (Figure 2(a)). Short-chain fatty acids (C10–C12), which include lauric acid, are reported as mid-range inhibitors of COX-1 (about 50–60% of inhibition) and COX-2 (about 25–30% inhibition) in vitro [51].

Barbosa et al. [52] demonstrated that babassu oil decreased ischemia-induced macromolecular leakage in postcapillary venules and inhibited histamine-induced microvascular permeability increase in hamster cheek pouch. To verify if the same occurs in skin, instead of mucosa, ethyl phenylpropiolate-induced ear edema was performed. EPP administration immediately increases vascular permeability, with blood flow increasing somewhat slowly. Histamine, serotonin (5-HT), kinins, and prostaglandins combined actions on vascular permeability seemed to be involved in early EPP response [53]. Babassu oil (10 *μ*L/ear) and lauric acid (4 mg/ear) inhibited EPP-induced ear edema by 82.0 and 71.8% (*p* < 0.001), respectively, in accordance with the results of Barbosa et al. [52]. Dexamethasone (0.1 mg/ear), used as positive control, inhibited edema formation by 65.6% (*p* < 0.001) (Figure 2(b)).

Phenol-induced skin inflammation is very like human contact dermatitis processes [54]. Phenol disrupts the plasma membranes of keratinocytes in the skin, resulting in release of preformed cytokines (IL-1*α*, IL-8, and TNF-*α*). These cytokines stimulate the production of reactive oxygen species (ROS) and AA metabolites amplifying the inflammatory process [54–56]. Herein, babassu oil (10 *μ*L/ear) and lauric acid (4 mg/ear) inhibited phenol-induced ear edema by 89.8 and 90.0% (*p* < 0.001), respectively. Dexamethasone (0.1 mg/ear), used as positive control due to its membrane stabilizing effect, inhibited edema formation by 91.0% (*p* < 0.001) (Figure 2(c)).

Topical administration of capsaicin releases proinflammatory mediators such as substance P and histamine, due to TRPV-1 activation, which result in an immediate vasodilation and erythema followed by edema. Maximum edema is achieved within 30 minutes after capsaicin administration [57, 58]. Neither babassu oil (10 *μ*L/ear) nor lauric acid (4 mg/ear) was able to inhibit capsaicin-induced ear edema. Ruthenium red (3 mg/kg, s.c.), given 30 min before capsaicin administration, reduced the ear edema by 75.3% (*p* < 0.01) (data not shown).

### 3.3. HLB Value and Microemulsion Formulation

The HLB number is a semiempirical scale for selecting surfactants [26]. The corrected HLB of the selected surfactant or blend of surfactants that match the HLB of the selected oil provides the lowest interface tension between the oil and water phases and gives the system stability [28]. In this context, the most stable emulsion is obtained in system with the smallest droplet size. Analysis by DLS showed the smallest mean droplet diameters in babassu oil emulsions at an HLB of 8.0 (2.3882 *μ*m).

The maximum turbidity values are the same HLB value at which the mean droplet diameter is minimal [27]. Here, we found that the highest mean turbidity values for babassu oil emulsions were obtained at HLB values from 8.0 to 10.0 (0.8–1.06). The correlation coefficient (Pearson *r*) between the turbidity values and the mean droplet size for the emulsion with an HLB value of 8.0 was 0.275 (*r*^2^ = 0.075624), showing a positive correlation (*p* < 0.05, ANOVA followed by Tukey's test).

Using the HLB value required for the babassu oil (HLB 8.0), Span 80 and Kolliphor EL were selected as the surfactants at a ratio of 6 : 4, respectively, because this blend had an HLB value equal to that determined for the oil. The aqueous phase was composed of water and propylene glycol (1 : 3) and the oil phase was babassu oil. In most cases, single-chain surfactants alone are unable to reduce the interfacial tension sufficiently to form a microemulsion. Propylene glycol was added as a cosurfactant to decrease the interfacial tension and increase the fluidity of the interface. In this case, part of the propylene glycol content was incorporated into the surfactant layer, and the other part decreased the polarity of the water by dissolving in the water. However, a higher amount of propylene glycol molecules favors formation of a bicontinuous microemulsion and avoids rigid structures, such as gels and liquid-crystals [59].

**Figure 3:**
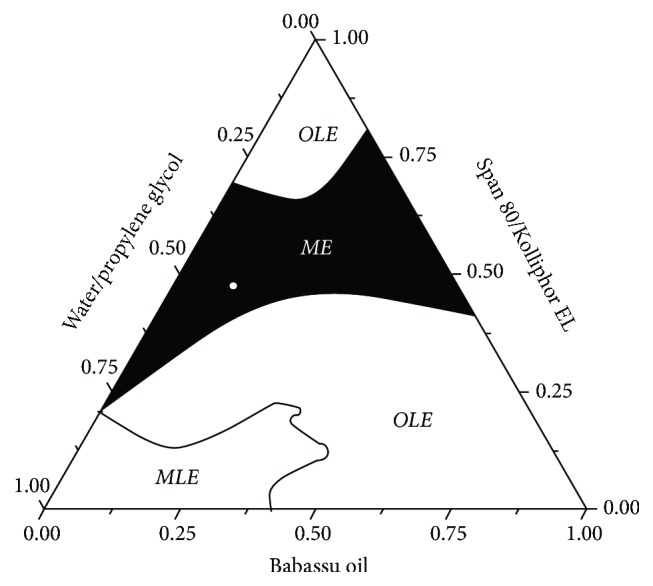
Pseudo-ternary phase diagram (8 : 2) of the babassu oil microemulsion. The black region includes the microemulsion systems (ME) and the selected formulation (white dot). OLE, opaque liquid emulsions; MLE, milk liquid emulsions.

The transparent liquid systems formed by the pseudo-ternary phase diagram can be used to obtain concentration ranges of babassu oil, emulsifiers, and the aqueous phase for microemulsion formulations. A system composed of 39% aqueous phase, 12.2% oil phase, and 48.8% surfactants (Figure 3, white dot) was selected for physical and morphological characterization. The selected system exhibited the typical characteristics of a microemulsion system, such as slight viscosity, transparency, and stability. In the diagram, this formulation is found near the borderline of o/w and bicontinuous microemulsions, which may indicate a transitional phase structure. In addition, the ratio of system components favors the formation of oil droplets surrounded by water, suitable for biological applications.

### 3.4. Electrical Conductivity

Electrical conductivity is commonly used to characterize the microstructure transitions that occur in microemulsions, that is, transformation from water-entrapped systems to intermediate structures and then to water continuous microstructures. Conductivity is low for reverse structures in nonconducting oil media that have little interactions with each other. When more water is added to the system, the conductive droplets begin to contact one another and form other structures, resulting in increased EC [60]. This phenomenon may have occurred in the babassu microemulsion because its conductivity value was 20.96 *μ*S/cm.

### 3.5. Morphological Analysis

**Figure 4:**
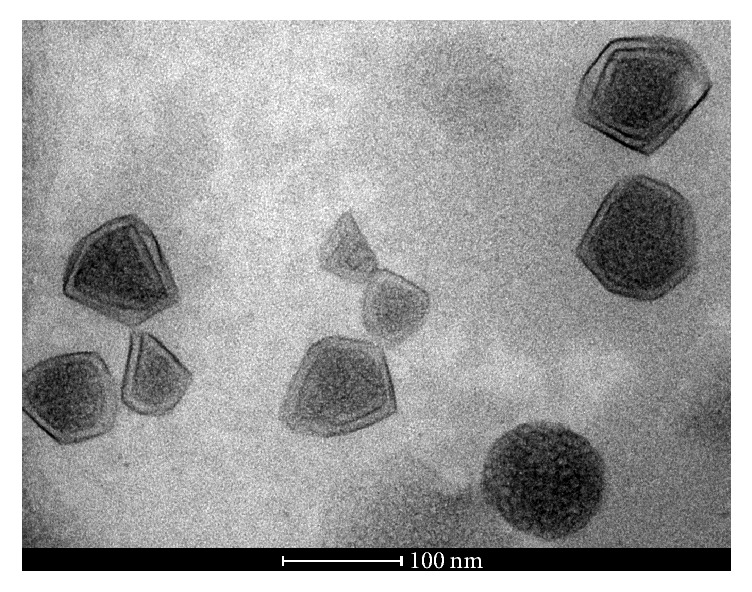
The ultrastructure of the babassu microemulsion that shows clusters of nanodroplets filled with oil, some of which have merged with one another and are surrounded by the surfactant interface and aqueous phase.

The ultrastructure of the babassu microemulsion was investigated using transmission electron microscopy (Figure 4). We observed clusters of nanodroplets filled with oil that were surrounded by an electron dense material composed of the surfactant interface bonded to the aqueous phase. Most of the oil droplets were connected side by side, but some of them were merging, indicating a transition to an o/w microemulsion. In addition, thick peripheral layers surrounding the nanodroplet network were sometimes observed, which could be related to displacement of the water and emulsifiers from the inner phase during system transformation.

The microemulsion composition and individual characteristics of components define the phase morphology of the system. As the concentration of water increases, the droplets increase in size and eventually form a cluster that is considered infinite. At this stage, the microemulsion possesses a bicontinuous structure. Further addition of the water phase transforms the bicontinuous system into an o/w microemulsion, where the droplets of the organic phase are surrounded by the water bath, and the interface is composed of surfactant species. During each of these transition steps, morphological phases in intermediate regions may be formed, and the stability of the system depends on thermodynamic conditions [60–62].

### 3.6. SAXS Measurements

SAXS is a well-established technique used to investigate the morphology, shape, and size of a multiphase sample, namely, aggregates dispersed in liquids, to obtain structural information regarding inhomogeneities based on the difference of electron density in the samples. This technique provides them with a characteristic length on the order of tens to hundreds of Angstroms (Å) [63, 64].

**Figure 5:**
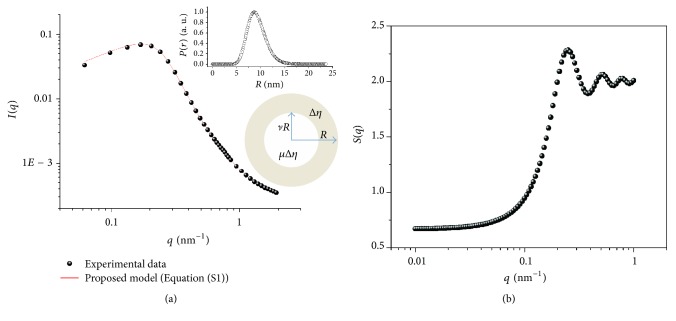
(a) Scattering intensity *I* of the SAXS measurements as a function of the scattering vector *q* for the babassu oil microemulsion. The solid line is the fit according to supplementary file procedure (Equation (S1)). The spherical shell had an average outer radius of *R* = 8.7 nm and inner radius of *vR* = 3.48 ± 0.02 nm. The normalized size distribution *P*(*r*) as a function of the overall radius (*R*) is also shown. (b) Structure factor *S*(*q*) for a hard sphere interaction potential of the babassu microemulsion with an average correlation distance (*R*_HS_) of 12 ± 0.01 nm and a volume fraction of 0.14 ± 0.001.

Figure 5(a) shows the experimental data simulated according to (3) (see SM). The proposed model fits the experimental data, which present droplets sizes from 5 to 15 nm with the majority size of approximately 8.7 nm.

The scattering length density difference between the shell and matrix (Δ*η*) for the microemulsion was 8.5 ± 0.03 nm, and the *ν* value was 0.4 ± 0.01. The SAXS results showed that the structure had a thin transition layer between the oil and aqueous phases. In this case, the surfactant hydrophobic chains interacted with babassu oil, which was observed as a small transition region that surrounded the oil droplets in the TEM micrograph (see Figure 4). *S*(*q*) is the Fourier transform of the pair correlation function, which in turn depends on the pair potential between micelles. However, the Percus-Yevick approximation provides an analytical expression for *S*(*q*) for monodisperse particles acting as hard spheres [65]. The structure factor was estimated using the monodisperse approach. This method only multiplies the size averaged form factor with the structure factor. The interaction potentials between the particles are assumed to be spherical, symmetric, and independent of the particle size [66, 67].  Figure 5(b) shows the average structure factor for the microemulsion sample. The peak at approximately *q* ~ 0.25 nm^−1^ in the *S*(*q*) curve indicates the correlation distance between the oil droplets. Besides, the other peaks at around *q* ~ 0.50 and 0.78 nm^−1^ show that the average interaction potential distance between the oil droplets was approximately 12 ± 0.01 nm.

### 3.7. DSC Analysis

**Figure 6:**
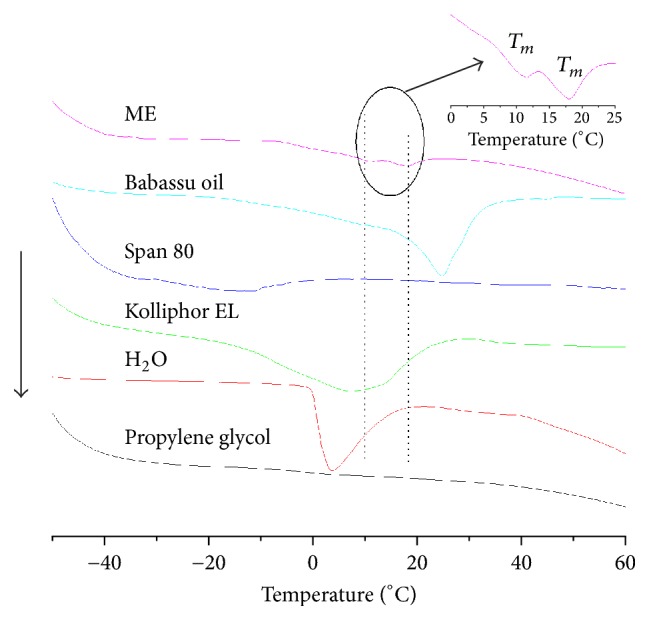
DSC thermographs for the babassu microemulsion, showing a broad endothermic melt transition in two temperature regions (inset).

The thermal behaviors of water in the microemulsion system were investigated by DSC and compared with that of pure water (Figure 6). Initially, the samples were cooled to −50°C and then heated to 60°C at a rate of 10°C/min. The thermal transition for the propylene glycol sample was not observed in the temperature range. Pure water showed an endothermic transition near 0°C and enthalpy of fusion of approximately 330 J/g [68]. Pure babassu oil showed two endothermic peaks at 7 and 25°C that were related to the melting temperatures of fatty acid components [69]. The multiple peaks that were observed for the oil sample are due to the polymorphism of different fatty acid. Most vegetable oils can be found in at least three crystalline forms, designated as *α*, *β*′, and *β* [70, 71]. The enthalpy of fusion of pure oil is approximately 100 ± 0.02 J/g, whereas the microemulsion sample showed a broad endothermic melt (fusion) transition in two temperature regions at 11 and 18°C (Figure 6, inset). It is essential to note that the presence of a nearby surface alters the thermodynamic properties, such as freezing point, melting point, and enthalpy [72, 73]. The endothermic transition was not observed at the same position as pure water. This effect is related to the partial miscibility of the surfactants and propylene glycol with water [74]. The literature has shown that the melting temperature deviation in relation to pure water depends on the nature of the interaction between the surfactant and water [72, 73, 75]. When the surfactant concentration is low, the transition of pure water is observed at approximately 0°C. Therefore, when we are dealing with systems in which the interactions depend on the concentrations, the surfactant properties can strongly contribute to the melting temperature and fusion enthalpy. For this reason, the microemulsion enthalpy decreased by 9.5 ± 0.2 J/g when compared with water and pure oil.

The presence of two melting peaks is a characteristic behavior of a bicontinuous microemulsion in situations where the oil phase is composed of a single fatty acid [60, 76–78]. However, when the oil phase consists of a triglyceride molecule, which is composed of various fatty acids, this behavior depends on the interactions of each fatty acid with the surfactants species. The first peak in the DSC thermogram of the microemulsion (~11°C) is related to the amount of free water. The second peak (~18°C) refers to the fusion of the oil phase in microemulsion. This thermal transition is shifted to lower temperatures because the fatty acids interact with the surfactant through the nonpolar head.

### 3.8. Rheological Behavior of the System

The rheological behavior of the babassu microemulsion is demonstrated in SM (Figure S5). The shear stress is proportional to increased shear rate, which is characteristic of Newtonian behavior. The profile of shear stress versus shear rate for Newtonian liquids yields a straight line, which is the expected behavior of microemulsions [79–81]. The average viscosity value for the babassu microemulsion was approximately 0.29 ± 0.1 Pas.

### 3.9. Topical Anti-Inflammatory Activity of Babassu Microemulsion

**Figure 7:**
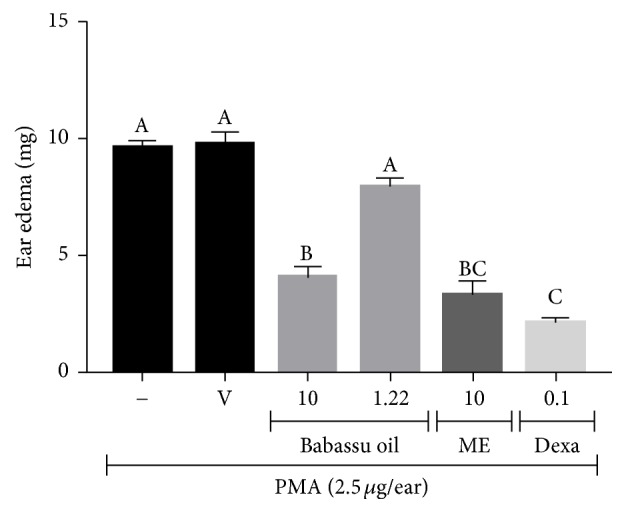
Topical anti-inflammatory activity of babassu oil and microemulsion in PMA-induced ear edema. Results are expressed as the mean ± SEM and analyzed by ANOVA followed by Tukey's test with *p* set at 0.05. Different letters show statistical differences between groups (*p* < 0.05). (−) Negative control (acetone); (V) microemulsion vehicle (48.8% surfactants, 39% aqueous phase, and 12.2% water); babassu oil (*μ*L/ear); (ME) babassu microemulsion (*μ*L/ear); and (Dexa) dexamethasone (mg/ear).

The topical anti-inflammatory activity of the babassu microemulsion compared with pure babassu oil is shown in Figure 7. Pure babassu oil and 12.2% babassu oil diluted in acetone inhibited the PMA-induced ear edema by 58.0% (*p* < 0.001) and 17.5% (not significant), respectively. The babassu microemulsion inhibited ear edema by 66.2% (*p* < 0.001), showing an enhanced activity promoted by the microemulsification of babassu oil, since babassu oil at 12.2% (final concentration in the microemulsion) did not have a significant effect on this experimental model. Dexamethasone (0.1 mg/ear) inhibited PMA-induced ear edema by 78.4% (*p* < 0.001).

Skin permeation enhancement by microemulsions has been widely studied for several anti-inflammatory drugs, for example, indomethacin, aspirin, and rofecoxib [82–84]. Microemulsification of babassu oil seems to enhance skin permeation of anti-inflammatory active compounds found in the oil, reaching the same percentages of edema inhibition as pure babassu oil with a much lower oil concentration.

## 4. Conclusions

In this work, we determined the chemical composition of the main fatty acids of babassu oil using GC-MS and the results were confirmed by ^1^H and ^13^C NMR spectra. Moreover, ^13^C NMR provided additional information, showing that triacylglycerol was the only positional fatty acid isomer in the glycerol backbone.

Babassu oil and lauric showed topical anti-inflammatory activity in different phlogistic agents-induced ear edema in mice, probably due to inhibition of AA metabolism and prostaglandin biosynthesis and/or action, release of histamine and serotonin, and inhibition of preformed cytokine release.

The developed babassu nanosystem was characterized as a phase transition microemulsion, which can be considered similarly bicontinuous as an o/w phase in contrast with classical microemulsion systems. Addition of more aqueous phase should form a well-defined o/w microemulsion.

The babassu oil microemulsion obtained here displayed advantages due to the combined features of bicontinuous and o/w microemulsions, including very low interfacial tension, high fluctuating interface, and high solubilizing properties. This system may have the ability to incorporate hydrophilic and/or lipophilic drugs that could be released faster than globular microemulsions with superior stability against the aqueous biological environment.

Topical anti-inflammatory activity of babassu oil was enhanced by microemulsification, reaching the same ear edema inhibition as pure babassu oil at a much lower concentration. This synthesized nanocarrier represents a new promising strategy for diseases treatment because babassu oil contains fatty acids with important biological properties, such as antioxidant, anti-inflammatory, antitumor, and antimicrobial.

## Figures and Tables

**Table 1:** Physicochemical parameters of babassu oil from Chapada do Araripe, Brazil.

Physicochemical parameters	Babassu oil (unrefined)	Reference value (refined oil)
Relative density (g/mL)	0.9210	0.9140–0.9170
Refractive index at 40°C	1.458	1.448–1.451
Acid value (mgKOH/g)	0.13	Max. 4
Peroxide value (meq/kg)	nd	Max. 15
Rancidity	Absent	Absent

nd: not detected.

**Table 2:** Fatty acid composition of babassu oil from Chapada do Araripe, Brazil.

Skeleton	Compound	Area (%) ± St Dev
C12:0	Dodecanoic acid	40.78 ± 1.56
C13:0	Tridecanoic acid	0.03 ± 0.01
C14:0	Tetradecanoic acid	20.05 ± 0.27
C16:0	Hexadecanoic acid	12.26 ± 0.59
C18:2n6c	(Z,Z)-9,12-Octadecadienoic acid	2.39 ± 0.29
C18:1n9c	(Z)-9-Octadecenoic acid	21.35 ± 0.36
C18:0	Octadecanoic acid	2.64 ± 0.09

**Table 3:** ^1^H and ^13^C NMR chemical shift (*δ*) data of babassu oil in CDCl_3_.

Hydrogen	*δ* (ppm)	Carbon	*δ* (ppm)
CH=CH	5.33	C=O	173.2 and 172.8
CH-O	5.22	CH=CH	129.9 and 129.6
CH_2_-O	4.29	CH_2_-O	62.1
CH_2_-O	4.15	CH-O	68.8
C=C-CH_2_-C=C	2.74	C=C-CH_2_-C=C	27.2
CH_2_-C=O	2.30	CH_2_-C=O	34.1
CH_2_-C=C	2.01	CH_2_-C=C	31.8
CH_2_-CH_2_-C=O	1.61	CH_2_-CH_2_-C=O	24.8
${\left({C\underline{H}}_{2}\right)}_{n}$	1.28	CH2-CH3	22.6
CH_3_	0.87	${\left(\underline{C}H_{2}\right)}_{n}$	29.7–28.8
		CH_3_	14.0

## Abbreviations

AA: Arachidonic acid
BA: Babassu oil
COX: Cyclooxygenase
DAG: Diacylglycerols
Dexa: Dexamethasone
DLS: Dynamic light scattering
DSC: Differential scanning calorimetry
EC: Electrical conductivity
EPP: Ethyl phenylpropiolate
GC-MS: Gas chromatography-mass spectrometry
HLB: Hydrophilic-lipophilic balance
IL: Interleukin
Indo: Indomethacin
LA: Lauric acid
LNLS: Brazilian synchrotron light laboratory
MAG: Monoacylglycerols
ME: Microemulsion
NMR: Nuclear magnetic resonance
O/W: Oil-in-water
PG: Prostaglandin
PLA2: Phospholipase A2
PMA: Phorbol 12-myristate 13-acetate
ROS: Reactive oxygen species
SAXS: Small-angle X-ray scattering
TAG: Triacylglycerols
TEM: Transmission electron microscopy
TMS: Tetramethylsilane
TNF: Tumor necrosis factor
W/O: Water-in-oil.

## Supplementary Materials

Supplementary Materials As supplementary material one can find the GC-MS chromatogram and also H1 and C13 MNR spectra of babassu oil. Data regarding droplet size distribution, shear stress, and stability studies can also be found. SAXS equations used in the measurement of babassu microemulsion are described in detail in this file. Fig. S1: droplet size and turbidity as a function of HLB. The values from 8 to 10 showed the smallest droplet size and the highest turbidity, which is indicative of more stable emulsions. Fig. S2: GC-MS chromatogram of the babassu oil fatty acids. Fig. S3: 1H NMR spectrum of the babassu oil sample in CDCl3. Fig. S4: 13C NMR spectrum of the babassu oil sample in CDCl3. Fig. S5: microemulsion rheological curve showing Newtonian behavior. Table S1: centrifugation study to investigate the stability of Babassu microemulsion. Table S2: heating stress applied to babassu microemulsion to check stability in the temperature range 40–80°C. Table S3: heating-cooling cycles to check the effect of temperature variations on the stability of babassu microemulsion.

## References

[1] de Almeida G. M. A., Ramos M. A., Araújo E. L., Baldauf C., Albuquerque U. P. (2016). Human perceptions of landscape change: The case of a monodominant forest of Attalea speciosa Mart ex. Spreng (Northeast Brazil). *AMBIO*.

[2] Porro N., Veiga I., Mota D. (2011). Traditional communities in the Brazilian Amazon and the emergence of new political identities: The struggle of the quebradeiras de coco babaçu-babassu breaker women. *Journal of Cultural Geography*.

[3] Souza M. H. S. L., Monteiro C. A., Figueredo P. M. S., Nascimento F. R. F., Guerra R. N. M. (2011). Ethnopharmacological use of babassu (*Orbignya phalerata* Mart) in communities of babassu nut breakers in Maranhão, Brazil. *Journal of Ethnopharmacology*.

[4] Araújo F. R., González-Pérez S. E., Lopes M. A., Viégas I. D. J. M. (2016). Ethnobotany of babassu palm (Attalea speciosa Mart.) in the Tucuruí Lake Protected Areas Mosaic - Eastern Amazon. *Acta Botanica Brasilica*.

[5] Almeida Campos J. L., da Silva T. L. L., Albuquerque U. P., Peroni N., Lima Araújo E. (2015). Knowledge, Use, and Management of the Babassu Palm (Attalea speciosa Mart. ex Spreng) in the Araripe Region (Northeastern Brazil). *Economic Botany*.

[6] Mors W., T Rizzini C., Pereira N. A. (2000). *Medicinal Plants of Brazil*.

[7] Bieski I. G. C., Leonti M., Arnason J. T. (2015). Ethnobotanical study of medicinal plants by population of Valley of Juruena Region, Legal Amazon, Mato Grosso, Brazil. *Journal of Ethnopharmacology*.

[8] Miles E. A., Zoubouli P., Calder P. C. (2005). Differential anti-inflammatory effects of phenolic compounds from extra virgin olive oil identified in human whole blood cultures. *Nutrition Journal *.

[9] Cicerale S., Lucas L. J., Keast R. S. J. (2012). Antimicrobial, antioxidant and anti-inflammatory phenolic activities in extra virgin olive oil. *Current Opinion in Biotechnology*.

[10] Wu S.-J., Liu P.-L., Ng L.-T. (2008). Tocotrienol-rich fraction of palm oil exhibits anti-inflammatory property by suppressing the expression of inflammatory mediators in human monocytic cells. *Molecular Nutrition & Food Research*.

[11] Intahphuak S., Khonsung P., Panthong A. (2010). Anti-inflammatory, analgesic, and antipyretic activities of virgin coconut oil. *Pharmaceutical Biology*.

[12] de Sousa V. P., Crean J., Borges V. R. D. A. (2013). Nanostructured systems containing babassu (Orbignya speciosa) oil as a potential alternative therapy for benign prostatic hyperplasia. *International Journal of Nanomedicine*.

[13] Pessoa R. S., França E. L., Ribeiro E. B. (2014). Microemulsion of babassu oil as a natural product to improve human immune system function. *Drug Design, Development and Therapy*.

[14] Lawrence M. J., Rees G. D. (2012). Microemulsion-based media as novel drug delivery systems. *Advanced Drug Delivery Reviews*.

[15] Fanun M. (2012). Microemulsions as delivery systems. *Current Opinion in Colloid & Interface Science *.

[16] Naoui W., Bolzinger M.-A., Fenet B. (2011). Microemulsion microstructure influences the skin delivery of an hydrophilic drug. *Pharmaceutical Research*.

[17] Xavier-Junior F. H., Vauthier C., Morais A. R. V., Alencar E. N., Egito E. S. T. (2017). Microemulsion systems containing bioactive natural oils: an overview on the state of the art. *Drug Development and Industrial Pharmacy*.

[18] Mezni F., Maaroufi A., Msallem M., et al (2012). Fatty acid composition, antioxidant and antibacterial activities of Pistacia lentiscus L. fruit oils. *Journal of Medicinal Plants Research*.

[19] American Oil Chemists’ Society (1990). *Official Methods and Recommended Practices of the American Oil Chemists*.

[20] Metcalfe L. D., Pelka J. R., Schmitz A. A. (1966). Rapid preparation of fatty acid esters from lipids for gas chromatographic analysis. *Analytical Chemistry*.

[21] Milinsk M. C., Matsushita M., Visentainer J. V., De Oliveira C. C., De Souza N. E. (2008). Comparative analysis of eight esterification methods in the quantitative determination of vegetable oil fatty acid methyl esters (FAME). *Journal of the Brazilian Chemical Society*.

[22] Tubaro A., Dri P., Delbello G., Zilli C., Loggia R. D. (1986). The Croton oil ear test revisited. *Agents and Actions Supplements*.

[23] Carlson R. P., O'Neill-Davis L., Chang J., Lewis A. J. (1985). Modulation of mouse ear edema by cyclooxygenase and lipoxygenase inhibitors and other pharmacologic agents. *Agents and Actions Supplements*.

[24] Brattsand R., Thalén A., Roempke K., Källström L., Gruvstad E. (1982). Influence of 16*α*,17*α*-acetal substitution and steroid nucleus fluorination on the topical to systemic activity ratio of glucocorticoids. *The Journal of Steroid Biochemistry and Molecular Biology*.

[25] Gábor M., Rázga Z. (1992). Development and inhibition of mouse ear oedema induced with capsaicin. *Agents and Actions Supplements*.

[26] Griffin W. C. (1949). Classification of Surface Active Agents by HLB. *Journal of the Society of Cosmetic Chemists*.

[27] Orafidiya L. O., Oladimeji F. A. (2002). Determination of the required HLB values of some essential oils. *International Journal of Pharmaceutics*.

[28] Mahdi E. S., Sakeena M. H., Abdulkarim M. F., Abdullah G. Z., Sattar M. A., Noor A. M. (2011). Effect of surfactant and surfactant blends on pseudoternary phase diagram behavior of newly synthesized palm kernel oil esters. *Drug Design, Development and Therapy*.

[29] Ferrari R. A., Soler M. P. (2015). Obtention and characterization of coconut babassu derivatives. *Scientia Agricola*.

[30] Jackson F. L., Longenecker H. E. (1944). The fatty acids and glycerides of babassu oil. *Oil & Soap*.

[31] Ferreira B. S., Faza L. P., Le Hyaric M. (2012). A comparison of the physicochemical properties and fatty acid composition of indaiá (Attalea dubia) and Babassu (Orbignya phalerata) oils. *The Scientific World Journal*.

[32] Barison A., da Silva C. W. P., Campos F. R., Simonelli F., Lenz C. A., Ferreira A. G. (2010). A simple methodology for the determination of fatty acid composition in edible oils through 1H NMR spectroscopy. *Magnetic Resonance in Chemistry*.

[33] Gunstone F. D. (1993). *Advances in Lipid Methodology*.

[34] Knothe G., Kenar J. A. (2004). Determination of the fatty acid profile by 1H-NMR spectroscopy. *European Journal of Lipid Science and Technology*.

[35] Rosa A., Rescigno A., Piras A. (2012). Chemical composition and effect on intestinal Caco-2 cell viability and lipid profile of fixed oil from Cynomorium coccineum L.. *Food and Chemical Toxicology*.

[36] Sacchi R., Addeo F., Giudicianni I. (1992). Analysis of the positional distribution of fatty acids in olive oil triacylglycerols by high resolution 13C-NMR of the carnonyl region. *Italian Journal of Food Science*.

[37] Medeiros R., Otuki M. F., Avellar M. C. W., Calixto J. B. (2007). Mechanisms underlying the inhibitory actions of the pentacyclic triterpene *α*-amyrin in the mouse skin inflammation induced by phorbol ester 12-*O*-tetradecanoylphorbol-13-acetate. *European Journal of Pharmacology*.

[38] Murakawa M., Yamaoka K., Tanaka Y., Fukuda Y. (2006). Involvement of tumor necrosis factor (TNF)-*α* in phorbol ester 12-O-tetradecanoylphorbol-13-acetate (TPA)-induced skin edema in mice. *Biochemical Pharmacology*.

[39] Shin Y. H., Yoon S.-H., Choe E.-Y. (2007). PMA-induced up-regulation of MMP-9 is regulated by a PKCa-NF-?B cascade in human lung epithelial cells. *Experimental Molecular Medicine*.

[40] Alves I. A. B. S., Santos S. M., Mendes R. F. V. (2017). Chemical composition, antioxidant and topical anti-inflammatory activities of Croton cordiifolius Baill. (Euphorbiaceae). *Journal of Medicinal Plant Research*.

[41] Bralley E. E., Greenspan P., Hargrove J. L., Wicker L., Hartle D. K. (2008). Topical anti-inflammatory activity of *Polygonum cuspidatum* extract in the TPA model of mouse ear inflammation. *Journal of Inflammation*.

[42] Rao T. S., Currie J. L., Shaffer A. F., Isakson P. C. (1993). Comparative evaluation of arachidonic acid (AA)- and tetradecanoylphorbol acetate (TPA)-induced dermal inflammation. *Inflammation*.

[43] Fischer C. L., Blanchette D. R., Brogden K. A. (2014). The roles of cutaneous lipids in host defense. *Biochimica et Biophysica Acta (BBA) - Molecular and Cell Biology of Lipids*.

[44] Nakatsuji T., Kao M. C., Fang J.-Y. (2009). Antimicrobial property of lauric acid against propionibacterium acnes: Its therapeutic potential for inflammatory acne vulgaris. *Journal of Investigative Dermatology*.

[45] Yang D., Pornpattananangkul D., Nakatsuji T. (2009). The antimicrobial activity of liposomal lauric acids against Propionibacterium acnes. *Biomaterials*.

[46] Huang W.-C., Tsai T.-H., Chuang L.-T., Li Y.-Y., Zouboulis C. C., Tsai P.-J. (2014). Anti-bacterial and anti-inflammatory properties of capric acid against Propionibacterium acnes: A comparative study with lauric acid. *Journal of Dermatological Science*.

[47] Kezutyte T., Desbenoit N., Brunelle A., Briedis V. (2013). Studying the penetration of fatty acids into human skin by ex vivo TOF-SIMS imaging. *Biointerphases*.

[48] Crummey A., Harper G. P., Boyle E. A., Mangan F. R. (1987). Inhibition of arachidonic acid-induced ear oedema as a model for assessing topical anti-inflammatory compounds. *Agents and Actions Supplements*.

[49] Opas E. E., Bonney R. J., Humes J. L. (1985). Prostaglandin and leukotriene synthesis in mouse ears inflamed by arachidonic acid. *Journal of Investigative Dermatology*.

[50] Humes J. L., Opas E. E., Galavage M., Soderman D., Bonney R. J. (1986). Regulation of macrophage eicosanoid production by hydroperoxy- and hydroxy-eicosatetraenoic acids. *Biochemical Journal*.

[51] Henry G. E., Momin R. A., Nair M. G., Dewitt D. L. (2002). Antioxidant and cyclooxygenase activities of fatty acids found in food. *Journal of Agricultural and Food Chemistry*.

[52] Barbosa M. D. C. L., Bouskela E., Cyrino F. Z. (2012). Effects of babassu nut oil on ischemia/reperfusion-induced leukocyte adhesion and macromolecular leakage in the microcirculation: Observation in the hamster cheek pouch. *Lipids in Health and Disease*.

[53] Patrick E., Burkhalter A., Maibach H. I. (1987). Recent Investigations of Mechanisms of Chemically Induced Skin Irritation in Laboratory Mice.. *Journal of Investigative Dermatology*.

[54] Lim H., Park H., Kim H. P. (2004). Inhibition of contact dermatitis in animal models and suppression of proinflammatory gene expression by topically applied flavonoid, wogonin. *Archives of Pharmacal Research*.

[55] Murray A. R., Kisin E., Castranova V., Kommineni C., Gunther M. R., Shvedova A. A. (2007). Phenol-induced in vivo oxidative stress in skin: Evidence for enhanced free radical generation, thiol oxidation, and antioxidant depletion. *Chemical Research in Toxicology*.

[56] Wilmer J. L., Burleson F. G., Kayama F., Kanno J., Luster M. I. (1994). Cytokine induction in human epidermal keratinocytes exposed to contact irritants and its relation to chemical-induced inflammation in mouse skin. *Journal of Investigative Dermatology*.

[57] Gábor M. (2000). *Mouse Ear Inflammation Models and their Pharmacological Applications*.

[58] Zegarska B., Leliñska A., Tyrakowsk T. (2006). Clinical and experimental aspects of cutaneous neurogenic inflammation. *Pharmacological Reports*.

[59] Szumała P. (2015). Structure of Microemulsion Formulated with Monoacylglycerols in the Presence of Polyols and Ethanol. *Journal of Surfactants and Detergents*.

[60] Kogan A., Shalev D. E., Raviv U., Aserin A., Garti N. (2009). Formation and characterization of ordered bicontinuous microemulsions. *The Journal of Physical Chemistry B*.

[61] Giannakas A. E., Vaimakis T. C., Ladavos A. K., Trikalitis P. N., Pomonis P. J. (2003). Variation of surface properties and textural features of spinel ZnAl2O4 and perovskite LaMnO3 nanoparticles prepared via CTAB-butanol-octane-nitrate salt microemulsions in the reverse and bicontinuous states. *Journal of Colloid and Interface Science*.

[62] Hayes D. G., Gomez Del Rio J. A., Ye R., Urban V. S., Pingali S. V., O'Neill H. M. (2015). Effect of protein incorporation on the nanostructure of the bicontinuous microemulsion phase of winsor-III systems: A small-angle neutron scattering study. *Langmuir*.

[63] Bianchi O., Barbosa L. G., MacHado G., Canto L. B., Mauler R. S., Oliveira R. V. B. (2013). Reactive melt blending of PS-POSS hybrid nanocomposites. *Journal of Applied Polymer Science*.

[64] Mota A. A. R., Gatto C. C., Machado G. (2014). Structural organization and supramolecular interactions of the task-specific ionic liquid 1-methyl-3-carboxymethylimidazolium chloride: Solid, solution, and gas phase structures. *The Journal of Physical Chemistry C*.

[65] Percus J. K., Yevick G. J. (1958). Analysis of Classical Statistical Mechanics by Means of Collective Coordinates. *Physical Review A: Atomic, Molecular and Optical Physics*.

[66] Glatter O., Kratky O., first. (1982). *Small Angle X-Ray Scattering*.

[67] Svergun D. I. (1987). *Structure Analysis by Small-Angle X-Ray and Neutron Scattering*.

[68] Atkins P., Paula J. (2012). *Elements of Physical Chemistry*.

[69] Nassu R. T., Gonçalves L. A. G. (1999). Determination of melting point of vegetable oils and fats by differential scanning calorimetry (DSC) technique. *Grasas y Aceites*.

[70] O'Brien R. D. (2008). *Fats and Oils: Formulating and Processing for Applications*.

[71] Wang T., Briggs J. L. (2002). Rheological and thermal properties of soybean oils with modified FA compositions. *Journal of the American Oil Chemists’ Society*.

[72] Garti N., Aserin A., Tiunova I., Fanun M. (2000). A DSC study of water behavior in water-in-oil microemulsions stabilized by sucrose esters and butanol. *Colloids and Surfaces A: Physicochemical and Engineering Aspects*.

[73] Podlogar F., Gašperlin M., Tomšič M., Jamnik A., Rogač M. B. (2004). Structural characterisation of water-Tween 40/Imwitor 308-isopropyl myristate microemulsions using different experimental methods. *International Journal of Pharmaceutics*.

[74] Sardari F., Jouyban A. (2013). Solubility of nifedipine in ethanol + water and propylene glycol + water mixtures at 293.2 to 313.2 K. *Industrial & Engineering Chemistry Research*.

[75] Boonme P., Krauel K., Graf A., Rades T., Junyaprasert V. B. (2006). Characterization of microemulsion structures in the pseudoternary phase diagram of isopropyl palmitate/water/Brij 97:1-butanol. *AAPS PharmSciTech*.

[76] Fisher S., Wachtel E. J., Aserin A., Garti N. (2013). Solubilization of simvastatin and phytosterols in a dilutable microemulsion system. *Colloids and Surfaces B: Biointerfaces*.

[77] Hathout R. M., Woodman T. J., Mansour S., Mortada N. D., Geneidi A. S., Guy R. H. (2010). Microemulsion formulations for the transdermal delivery of testosterone. *European Journal of Pharmaceutical Sciences*.

[78] Note C., Koetz J., Kosmella S. (2006). Structural changes in poly(ethyleneimine) modified microemulsion. *Journal of Colloid and Interface Science*.

[79] Barnes H. A., Hutton J. F., Walters K. (1989). *An Introduction to Rheology*.

[80] Macosko C. W. (1994). *Rheology: principles, measurements, and applications*.

[81] Mouri A., Diat O., El Ghzaoui A. (2014). Phase behavior of reverse microemulsions based on Peceol®. *Journal of Colloid and Interface Science*.

[82] Barakat N., Fouad E., Elmedany A. (2011). Enhancement of skin permeation and anti-inflammatory effect of indomethacin using microemulsion. *Asian Journal of Pharmaceutics*.

[83] Desai K. G. H. (2004). Enhanced skin permeation of rofecoxib using topical microemulsion gel. *Drug Development Research*.

[84] Subramanian B., Kuo F., Ada E. (2008). Enhancement of anti-inflammatory property of aspirin in mice by a nano-emulsion preparation. *International Immunopharmacology*.

