# Supplementary material for: Anti-Inflammatory Activity of Babassu Oil and Development of a Microemulsion System for Topical Delivery
Source: Evid Based Complement Alternat Med. 2017 Dec 21;2017:3647801. doi: 10.1155/2017/3647801 (PMC5753019; doi:10.1155/2017/3647801)
Supplement: Supplementary Materials — As supplementary material one can find the GC-MS chromatogram and also H1 and C13 MNR spectra of babassu oil. Data regarding droplet size distribution, shear stress, and stability studies can also be found. SAXS equations used in the measurement of babassu microemulsion are described in detail in this file. Fig. S1: droplet size and turbidity as a function of HLB. The values from 8 to 10 showed the smallest droplet size and the highest turbidity, which is indicative of more stable emulsions. Fig. S2: GC-MS chromatogram of the babassu oil fatty acids. Fig. S3: 1H NMR spectrum of the babassu oil sample in CDCl3. Fig. S4: 13C NMR spectrum of the babassu oil sample in CDCl3. Fig. S5: microemulsion rheological curve showing Newtonian behavior. Table S1: centrifugation study to investigate the stability of Babassu microemulsion. Table S2: heating stress applied to babassu microemulsion to check stability in the temperature range 40–80°C. Table S3: heating-cooling cycles to check the effect of temperature variations on the stability of babassu microemulsion. [file 3647801.f1.pdf]

Supplementary Material

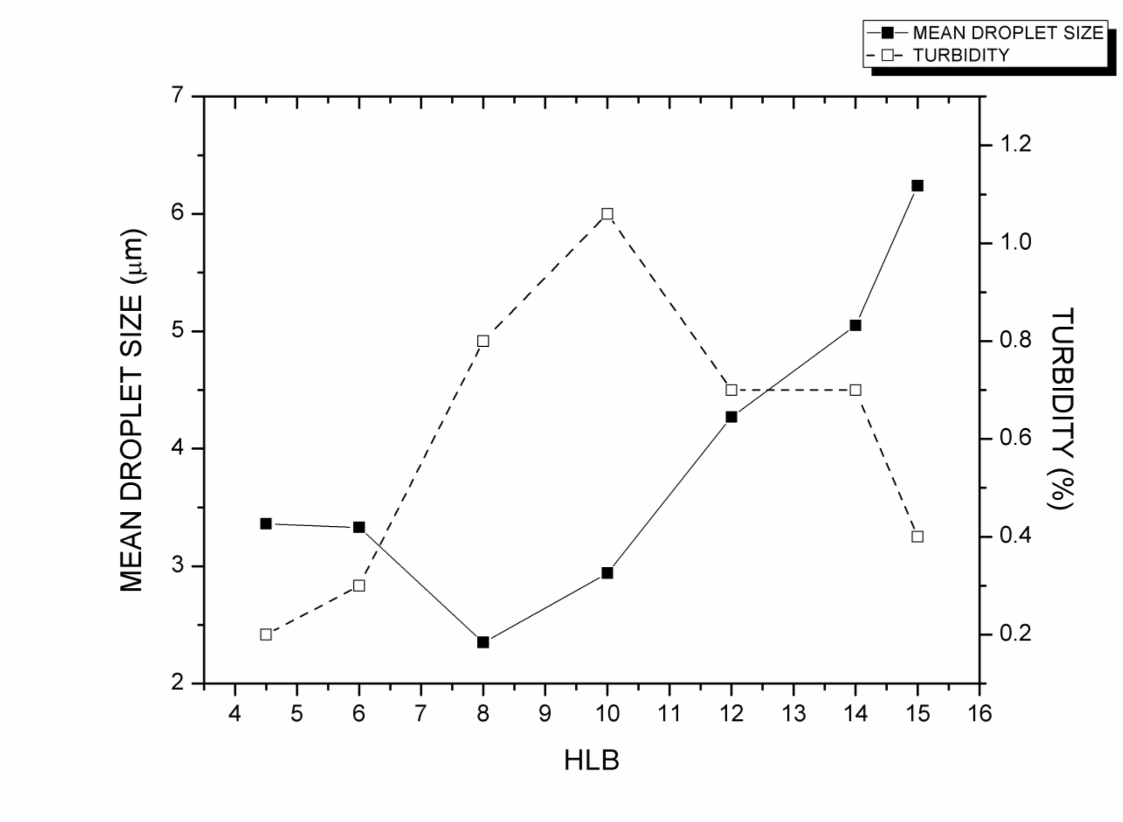

Fig. S1. Droplet size and turbidity as a function of HLB. The values from 8 to 10 showed the smallest droplet size and the highest turbidity, which is indicative of more stable emulsions.

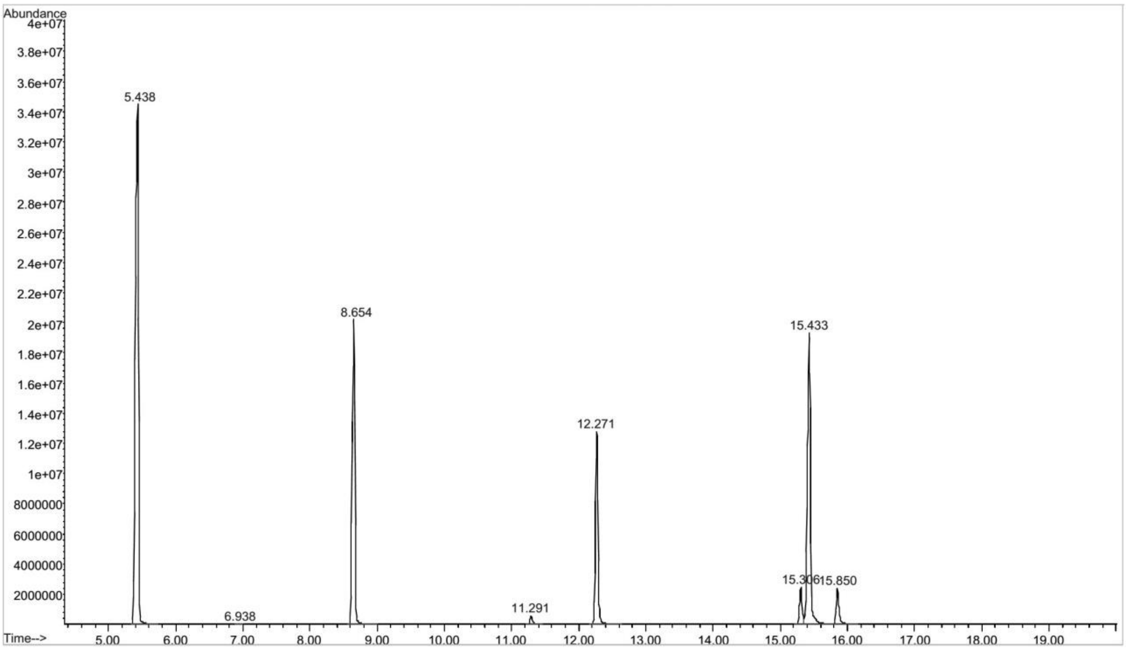

Fig. S2. GC-MS chromatogram of the babassu oil fatty acids.

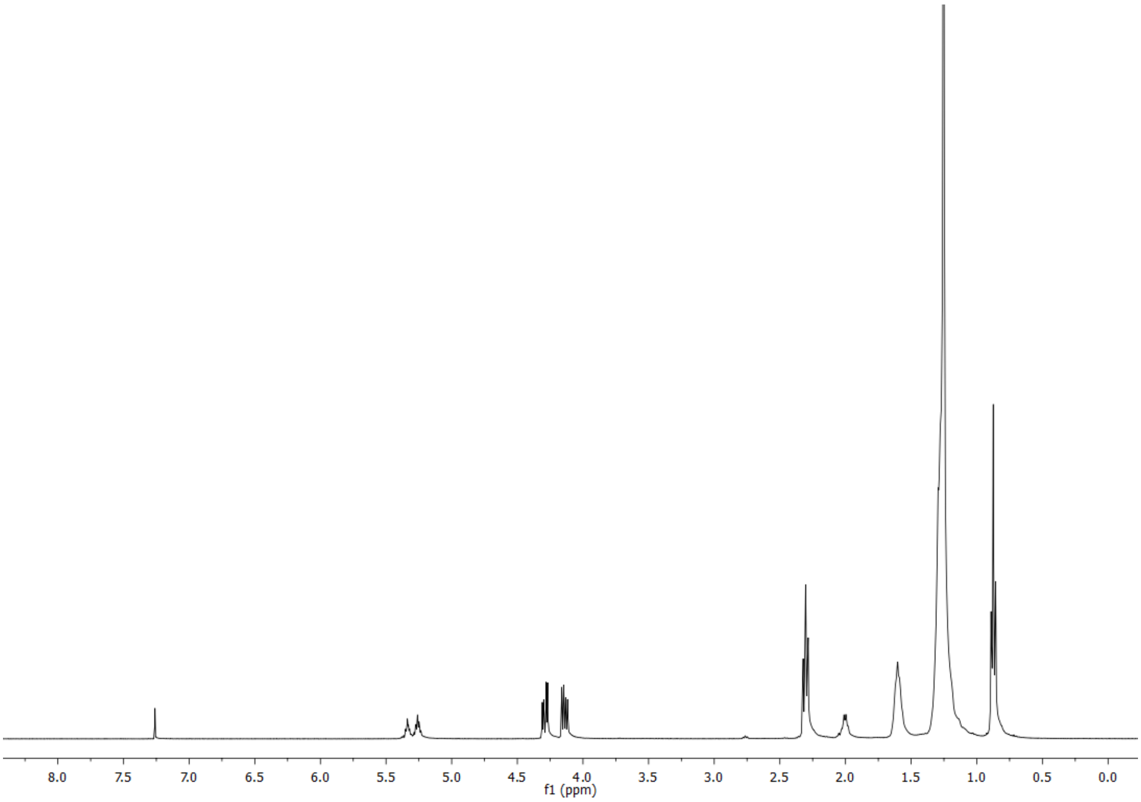

Fig. S3. ^{1}H NMR spectrum of the babassu oil sample in $CDCl_3$.

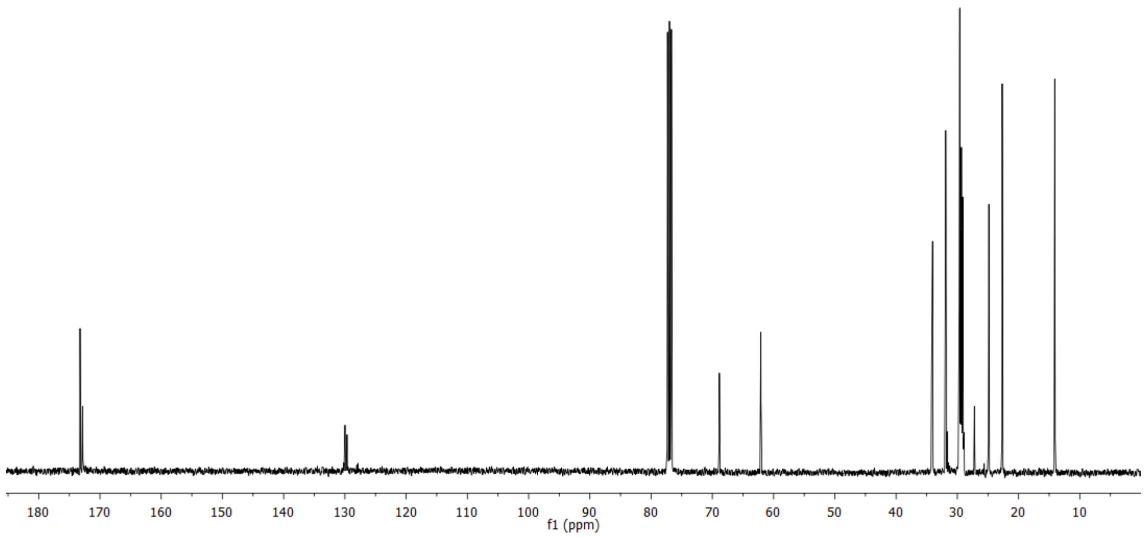

Fig. S4. ^{13}C NMR spectrum of the babassu oil sample in $CDCl_3$.

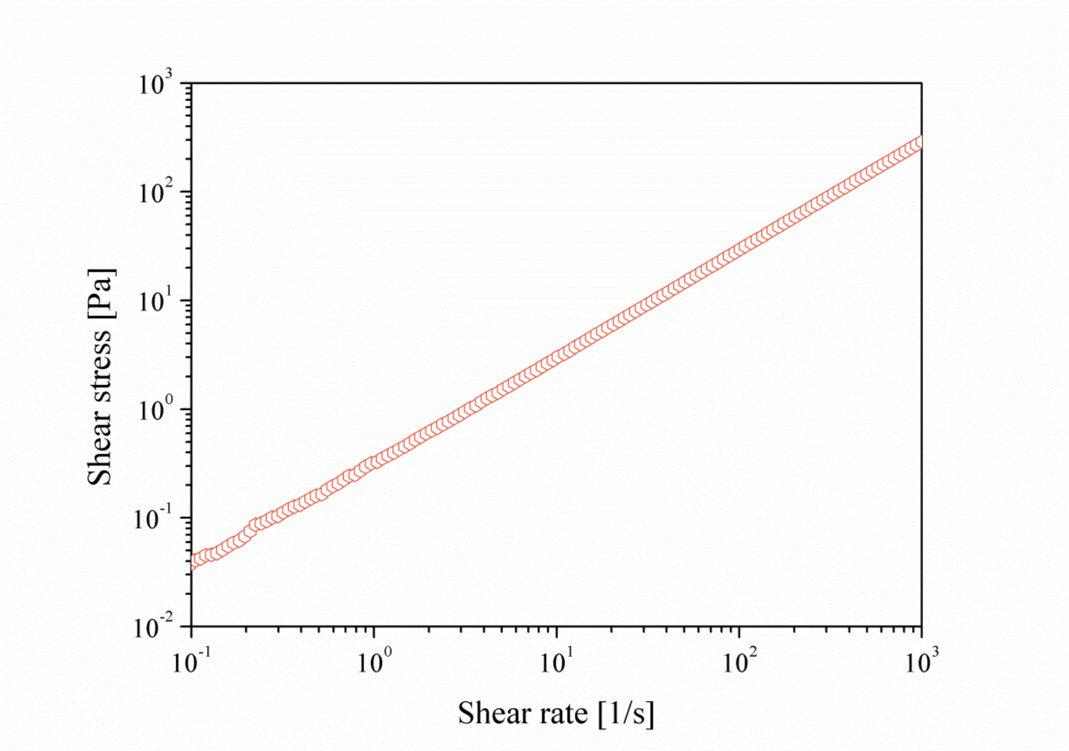

Fig. S5. Microemulsion rheological curve showing Newtonian behavior.

Table S1. Centrifugation study to investigate the stability of Babassu microemulsion.

Parameter	Before centrifugation[1]	After centrifugation[1]
pH[2]	6.7	7.13
Conductivity[3] ($\mu S.cm^{-1}$)	19.67	21.59
Droplet size[4] (nm)	48.64	50.68

[1]The centrifugation study was carried out at 15000 rpm for 30 min (NI 1801 centrifuge, Nova Instruments, Brazil).

[2]mPA210, Tecnopon, Brazil.

[3]mCA150, Tecnopon, Brazil.

[4]ZEN3690, Malvern instruments, United Kingdom.

Table S2. Heating stress applied to babassu microemulsion to check stability in the temperature range 40-80 °C.

Parameter	Before stress	40 °C	50 °C	60 °C	70 °C	80 °C
pH[1]	6.7	6.7	-	-	-	6.4
Conductivity[2] ($\mu S.cm^{-1}$)	19.67	0.69	-	-	-	1.12
Droplet size[3] (nm)	48.64	50.94	-	-	-	50.94
Instability (cracking, creaming and phase separation)	Absence					

[1]mPA210, Tecnopon, Brazil.

[2]mCA150, Tecnopon, Brazil.

[3]ZEN3690, Malvern instruments, United Kingdom.

Table S3. Heating–cooling cycles to check the effect of temperature variations on the stability of babassu microemulsion.

**Cycles* 1, 2, 3 and 4**	
4 °C	Frozen microemulsion
40 °C	No instability

*Heating–cooling cycles for a period of 24h.

SAXS equations

The intensity I(q) of the microemulsion can be analyzed according to a general expression as follows [1,2]:

$$I(q) \propto \langle F(q) \rangle^2 S(q) + \langle F(q)^2 \rangle - \langle F(q) \rangle^2 + I_{bg} \quad \text{(S1)}$$

where I_{bg} is the background intensity, *F(q)* is the form factor and *S(q)* is the structure factor. $\langle F(q) \rangle$ describes the polydisperse shape of the droplets, where the average is determined using a Lognormal distribution.

A form factor of the spherical core-shell that is separated by a correlation distance (R_{HS}) can be adopted. This form factor is parameterized with an outer radius R and an inner radius νR. The scattering contrast relative to the matrix of the core was μΔη and the difference between the matrix and the shell was Δη [1,2].

$$F(q,R,\nu,\Delta\eta,\mu) = (K(q,R,\Delta\eta) - K(q,\nu R,\Delta\eta(1-\mu)))^2 \quad \text{(S2)}$$

with

$$K(q,R,\Delta\eta)=\frac{4}{3}\pi R^3\Delta\eta 3\frac{\sin qR-qR\cos qR}{(qR)^3} \quad \text{(S3)}$$

In equations S2 and S3, R is the overall radius of the spherical shell, ν is the fraction of the overall radius, Δη is the scattering length density difference between the shell and matrix and μ is the scattering length density difference between the core and matrix relative to the shell contrast (Fig. 5 (a)). S(q) describes the spatial correlation between the droplets. In this work was used the hard sphere function to estimate the correlation length between droplets. The hard sphere function has been successfully used in many microemulsion studies [3-5]. However, R_{HS} is the independent radius of the core and can be interpreted as the interaction radius [6]. Experimental SAXS data were fitted using the software SASFIT® [7].

References

[1] D.I. Svergun, Structure Analysis by Small-Angle X-Ray and Neutron Scattering, first ed., Plenum Press, New York, 1987.

[2] O. Glatter, O. Kratky, Small Angle X-ray Scattering, first ed., Academic Press Inc, London, 1982.

[3] R. Wipf, M. Kraska, T. Spehr, J. Nieberle, H. Frey, B. Stühn, Interaction between a water-in-oil microemulsion and a linear-dendritic poly(propylene oxide)-polyglycerol block copolymer, Soft Matter 7 (2011) 10879-10888.2.

[4] M. Nayeri, M. Zackrisson, J. Bergenholtz, Scattering Functions of Core−Shell-Struct ured Hard Spheres with Schulz-Distributed Radii, J. Phys. Chem. B 113 (2009) 8296-830.

[5] H. Bagger-Jörgensen, L. Coppola, K. Thuresson, O. Olsson, K. Mortensen, Phase Behavior, Microstructure, and Dynamics in a Nonionic Microemulsion on

Addition of Hydrophobically End-Capped Poly(ethylene oxide), Langmuir 13 (1997) 4204-4218.

[6] N.W. Ashcroft, J. Lekner, Structure and Resistivity of Liquid Metals. Phys. Review 145 (1966) 83-90.

[7] J. Kohlbrecher, SASfit: a program for fitting simple structural models to small angle scattering data. Paul Scherrer Institut, Laboratory for Neutron Scattering, 2008.
